# 1-Fluoro-3,3-dimethyl-1,3-dihydro-1λ^3^-benzo[*d*][1,2]iodoxole

**DOI:** 10.1107/S1600536812012822

**Published:** 2012-03-31

**Authors:** Claude Y. Legault, Julie Prévost

**Affiliations:** a Université de Sherbrooke, Département de chimie, 2500 boul. de l’Université, Sherbrooke, Québec, Canada J1K 2R1

## Abstract

The asymmetric unit of the title compound, C_9_H_10_FIO, contains two independent mol­ecules which are weakly bound by inter­molecular O⋯I inter­actions [3.046 (4) and 2.947 (4) Å]. The two covalent I—F bonds are slightly longer than the two I—O bonds.

## Related literature

For information on the chemistry of hypervalent compounds, see: Zhdankin & Stang (2002 ▶); Wirth (2005 ▶). For the synthesis and structural analysis of the bromo analog of the title compound, see: Braddock *et al.* (2006 ▶). For the synthesis and structural analysis of the chloro analog of the title compound, see: Amey & Martin (1979 ▶); Niedermann *et al.* (2010 ▶). For related information on the *trans* effect in hypervalent iodine compounds, see: Ochiai *et al.* (2006 ▶).
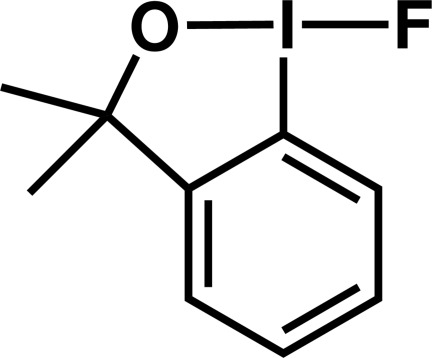


## Experimental

### 

#### Crystal data

C_9_H_10_FIO
*M* *_r_* = 280.07
Triclinic, 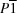

*a* = 7.983 (6) Å
*b* = 10.188 (8) Å
*c* = 11.691 (5) Å
α = 83.13 (5)°
β = 79.01 (5)°
γ = 78.27 (6)°
*V* = 910.6 (11) Å^3^
*Z* = 4
Mo *K*α radiation
μ = 3.48 mm^−1^
*T* = 193 K
0.4 × 0.4 × 0.3 mm

#### Data collection

Enraf–Nonius CAD-4 diffractometer
Absorption correction: ψ scan (*NRCVAX*; Gabe et al., 1989 ▶] *T*_min_ = 0.337, *T*_max_ = 0.422
3408 measured reflections
3408 independent reflections
2833 reflections with *I* > 2σ(*I*)
1 standard reflections every 100 reflections intensity decay: none

#### Refinement

*R*[*F* ^2^ > 2σ(*F* ^2^)] = 0.030
*wR*(*F* ^2^) = 0.073
*S* = 1.05
3408 reflections
217 parameters
H-atom parameters constrained
Δρ_max_ = 0.64 e Å^−3^
Δρ_min_ = −1.22 e Å^−3^

### 

Data collection: *DIFRAC* (Flack *et al.*, 1992 ▶); cell refinement: *DIFRAC*; data reduction: *NRCVAX* (Gabe *et al.*, 1989 ▶); program(s) used to solve structure: *SHELXS97* (Sheldrick, 2008 ▶); program(s) used to refine structure: *SHELXL97* (Sheldrick, 2008 ▶); molecular graphics: *Mercury* (Macrae *et al.*, 2006 ▶); software used to prepare material for publication: *publCIF* (Westrip, 2010 ▶).

## Supplementary Material

Crystal structure: contains datablock(s) I, global. DOI: 10.1107/S1600536812012822/lh5436sup1.cif

Supplementary material file. DOI: 10.1107/S1600536812012822/lh5436Isup2.cdx

Structure factors: contains datablock(s) I. DOI: 10.1107/S1600536812012822/lh5436Isup3.hkl

Supplementary material file. DOI: 10.1107/S1600536812012822/lh5436Isup4.cml

Additional supplementary materials:  crystallographic information; 3D view; checkCIF report

## Figures and Tables

**Table 1:** Selected bond lengths (Å)

C1—I1	2.085 (4)
C10—I2	2.094 (5)
F1—I1	2.045 (3)
F2—I2	2.046 (3)
I1—O1	2.022 (3)
I2—O2	2.017 (3)

**Fig. 1.**
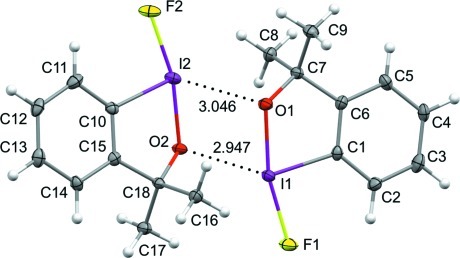
The asymmetric unit of the title compound, showing 30% probability displacement ellipsoids. The I···O interactions are shown by dotted lines. H atoms are depicted as circles of arbitrary size.

## supplementary crystallographic information

### Comment

Hypervalent iodine compounds have received a growing attention in recent years. This is not surprising considering that these reagents are polyvalent electrophiles and mild oxidants (Zhdankin & Stang, 2002; Wirth, 2005). In this family, haloiodanes are interesting yet under exploited electrophilic halogen sources. A research project currently underway in our group aims to exploit haloiodanes as electrophilic halogen sources. We developed a synthesis to obtain the title compound in order to evaluate and compare its reactivity with its chloro and bromo analogs. This is the first reported synthesis of the title compound.

In the crystal structure, two independent molecules, as shown in Fig. 1, are weakly bound by O···I interactions [3.046 (4) and 2.947 (4)Å]. The two I—O bonds observed measure 2.022 (3) Å and 2.017 (3) Å, respectively. These are shorter than the corresponding I—O bonds found in the chloro (2.042 (2) Å) (Amey & Martin, 1979; Niedermann *et al.*, 2010) and bromo (2.050 (5) Å) (Braddock *et al.*, 2006) analogs. This is consistent with the *trans* effect behavior described in a variety of hypervalent λ^3^-iodane compounds (Ochiai *et al.*, 2006). In contrast to the bromo analog, the title compound was found to be completely unreactive for the fluorination of anisole. While the title compound is a stable solid, caution must be taken when drying the crude solution. The use of anhydrous MgSO_4_ to dry the solution results in the displacement of the fluorine by a sulfate dianion. Drying by co-evaporation with benzene prevents this side reaction. A more in-depth study of the reactivity of this novel fluoroiodane is currently underway.

### Experimental

2-(2-Iodophenyl)-propan-2-ol (164 mg, 0.63 mmol) was dissolved in MeCN (3 ml) and SelectFluor (289 mg, 0.81 mmol) was added in one portion. The reaction was then stirred at room temperature for 16 h. The mixture was concentrated under reduced pressure. The crude product was dissolved in CH_2_Cl_2_ (10 ml), washed once with water (10 ml), and concentrated under reduced pressure. The crude product was dried by coevaporation with benzene. Crystals were grown by slow diffusion of a pentane solution on a CH_2_Cl_2_ solution of the title compound at room temperature.

### Refinement

The hydrogen atoms were placed at idealized calculated geometric positions and refined isotropically using a riding model.

### Crystal data

**Table:**

C_9_H_10_FIO	*Z* = 4
*M**_r_* = 280.07	*F*(000) = 536
Triclinic, *P*1	*D*_x_ = 2.043 Mg m^−^^3^
Hall symbol: -P 1	Mo *K*α radiation, λ = 0.71073 Å
*a* = 7.983 (6) Å	Cell parameters from 20 reflections
*b* = 10.188 (8) Å	θ = 10–12.5°
*c* = 11.691 (5) Å	µ = 3.48 mm^−^^1^
α = 83.13 (5)°	*T* = 193 K
β = 79.01 (5)°	Prism, white
γ = 78.27 (6)°	0.4 × 0.4 × 0.3 mm
*V* = 910.6 (11) Å^3^	

### Data collection

**Table:**

Enraf–Nonius CAD-4 diffractometer	*R*_int_ = 0
Graphite monochromator	θ_max_ = 25.6°, θ_min_ = 1.8°
ω scans	*h* = −9→9
Absorption correction: ψ scan (*NRCVAX*; Gabe et al., 1989]	*k* = 0→12
*T*_min_ = 0.337, *T*_max_ = 0.422	*l* = −13→14
3408 measured reflections	1 standard reflections every 100 reflections
3408 independent reflections	intensity decay: none
2833 reflections with *I* > 2σ(*I*)	

### Refinement

**Table:**

Refinement on *F*^2^	Primary atom site location: structure-invariant direct methods
Least-squares matrix: full	Secondary atom site location: difference Fourier map
*R*[*F*^2^ > 2σ(*F*^2^)] = 0.030	Hydrogen site location: inferred from neighbouring sites
*wR*(*F*^2^) = 0.073	H-atom parameters constrained
*S* = 1.05	*w* = 1/[σ^2^(*F*_o_^2^) + (0.0443*P*)^2^ + 0.462*P*] where *P* = (*F*_o_^2^ + 2*F*_c_^2^)/3
3408 reflections	(Δ/σ)_max_ = 0.001
217 parameters	Δρ_max_ = 0.64 e Å^−^^3^
0 restraints	Δρ_min_ = −1.22 e Å^−^^3^

### Special details

**Table:**

Geometry. All e.s.d.'s (except the e.s.d. in the dihedral angle between two l.s. planes) are estimated using the full covariance matrix. The cell e.s.d.'s are taken into account individually in the estimation of e.s.d.'s in distances, angles and torsion angles; correlations between e.s.d.'s in cell parameters are only used when they are defined by crystal symmetry. An approximate (isotropic) treatment of cell e.s.d.'s is used for estimating e.s.d.'s involving l.s. planes.
Refinement. The *DIFRAC*(Flack, 1992) program was used for centering, indexing, and data collection. One standard reflection was measured every 100 reflections, no decay was observed during data collection. The data were corrected for absorption by empirical methods based on psi scans and reduced with the *NRCVAX* (Gabe, 1989) programs. They were solved using *SHELXS97*(Sheldrick, 2008) and refined by full-matrix least squares on F2 with *SHELXL97*(Sheldrick, 2008). The non-hydrogen atoms were refined anisotropically. Refinement of *F*^2^ against ALL reflections. The weighted *R*-factor *wR* and goodness of fit *S* are based on *F*^2^, conventional *R*-factors *R* are based on *F*, with *F* set to zero for negative *F*^2^. The threshold expression of *F*^2^ > σ(*F*^2^) is used only for calculating *R*-factors(gt) *etc*. and is not relevant to the choice of reflections for refinement. *R*-factors based on *F*^2^ are statistically about twice as large as those based on *F*, and *R*- factors based on ALL data will be even larger.

### Fractional atomic coordinates and isotropic or equivalent isotropic displacement parameters (Å^2^)

**Table:**

	*x*	*y*	*z*	*U*_iso_*/*U*_eq_	
C1	0.5725 (6)	0.6621 (4)	0.4130 (4)	0.0211 (9)	
C2	0.7221 (6)	0.5801 (5)	0.4421 (4)	0.0272 (10)	
H2	0.8038	0.5305	0.3853	0.033*	
C3	0.7484 (7)	0.5727 (5)	0.5550 (5)	0.0339 (12)	
H3	0.849	0.5164	0.5777	0.041*	
C4	0.6295 (6)	0.6467 (5)	0.6364 (4)	0.0288 (11)	
H4	0.6489	0.6399	0.7148	0.035*	
C5	0.4833 (6)	0.7300 (5)	0.6056 (4)	0.0270 (10)	
H5	0.4045	0.7827	0.6617	0.032*	
C6	0.4513 (6)	0.7368 (4)	0.4917 (4)	0.0227 (9)	
C7	0.2966 (6)	0.8263 (4)	0.4478 (4)	0.0227 (9)	
C8	0.3279 (7)	0.9700 (5)	0.4218 (4)	0.0299 (11)	
H8A	0.2272	1.0273	0.3935	0.045*	
H8B	0.4316	0.9723	0.3618	0.045*	
H8C	0.3451	1.0031	0.4933	0.045*	
C9	0.1276 (6)	0.8183 (5)	0.5324 (4)	0.0309 (11)	
H9A	0.0311	0.8776	0.5008	0.046*	
H9B	0.1353	0.8467	0.6081	0.046*	
H9C	0.1077	0.7255	0.5426	0.046*	
C10	−0.0885 (6)	0.8468 (4)	0.0402 (4)	0.0215 (9)	
C11	−0.2429 (6)	0.9114 (5)	0.0057 (5)	0.0320 (12)	
H11	−0.3264	0.9704	0.0542	0.038*	
C12	−0.2712 (6)	0.8866 (5)	−0.1023 (5)	0.0308 (11)	
H12	−0.3758	0.9292	−0.1291	0.037*	
C13	−0.1484 (6)	0.8006 (5)	−0.1711 (4)	0.0302 (11)	
H13	−0.1687	0.7844	−0.2452	0.036*	
C14	0.0050 (6)	0.7373 (4)	−0.1332 (4)	0.0245 (10)	
H14	0.0887	0.678	−0.1814	0.029*	
C15	0.0367 (6)	0.7600 (4)	−0.0255 (4)	0.0212 (9)	
C16	0.1966 (6)	0.6918 (4)	0.0259 (4)	0.0204 (9)	
C17	0.3625 (6)	0.6881 (5)	−0.0644 (4)	0.0267 (10)	
H17A	0.4625	0.6434	−0.0281	0.04*	
H17B	0.3763	0.7802	−0.0937	0.04*	
H17C	0.3554	0.6384	−0.1296	0.04*	
C18	0.1750 (6)	0.5513 (4)	0.0787 (4)	0.0274 (10)	
H18A	0.2784	0.5075	0.1118	0.041*	
H18B	0.1601	0.4985	0.0178	0.041*	
H18C	0.0726	0.5574	0.1406	0.041*	
F1	0.7418 (4)	0.5802 (3)	0.1913 (3)	0.0372 (7)	
F2	−0.2633 (4)	0.9676 (3)	0.2491 (3)	0.0437 (8)	
I1	0.50006 (4)	0.68702 (3)	0.24847 (2)	0.02221 (10)	
I2	−0.01657 (4)	0.86419 (3)	0.20011 (2)	0.02531 (10)	
O1	0.2750 (4)	0.7761 (3)	0.3428 (3)	0.0283 (7)	
O2	0.2131 (4)	0.7722 (3)	0.1146 (3)	0.0250 (7)	

### Atomic displacement parameters (Å^2^)

**Table:**

	*U*^11^	*U*^22^	*U*^33^	*U*^12^	*U*^13^	*U*^23^
C1	0.025 (2)	0.018 (2)	0.022 (2)	−0.0066 (18)	−0.0071 (18)	0.0021 (17)
C2	0.022 (2)	0.027 (2)	0.031 (3)	0.000 (2)	−0.004 (2)	−0.0025 (19)
C3	0.031 (3)	0.032 (3)	0.041 (3)	−0.005 (2)	−0.018 (2)	0.005 (2)
C4	0.037 (3)	0.030 (3)	0.023 (2)	−0.010 (2)	−0.015 (2)	0.0036 (19)
C5	0.038 (3)	0.027 (2)	0.018 (2)	−0.012 (2)	−0.005 (2)	−0.0010 (18)
C6	0.024 (2)	0.020 (2)	0.024 (2)	−0.0023 (18)	−0.0066 (19)	0.0019 (17)
C7	0.022 (2)	0.025 (2)	0.020 (2)	−0.0004 (18)	−0.0024 (18)	−0.0050 (18)
C8	0.036 (3)	0.024 (2)	0.026 (2)	0.000 (2)	−0.003 (2)	0.0009 (19)
C9	0.027 (3)	0.034 (3)	0.027 (3)	−0.002 (2)	0.005 (2)	−0.003 (2)
C10	0.020 (2)	0.022 (2)	0.021 (2)	−0.0058 (18)	0.0004 (18)	−0.0009 (17)
C11	0.021 (2)	0.025 (2)	0.045 (3)	−0.001 (2)	0.000 (2)	0.002 (2)
C12	0.021 (2)	0.032 (3)	0.040 (3)	−0.007 (2)	−0.012 (2)	0.008 (2)
C13	0.033 (3)	0.027 (2)	0.034 (3)	−0.013 (2)	−0.012 (2)	0.007 (2)
C14	0.032 (3)	0.023 (2)	0.021 (2)	−0.009 (2)	−0.0069 (19)	−0.0009 (18)
C15	0.022 (2)	0.018 (2)	0.024 (2)	−0.0076 (18)	−0.0021 (18)	0.0011 (17)
C16	0.017 (2)	0.024 (2)	0.018 (2)	−0.0022 (18)	0.0009 (17)	−0.0054 (17)
C17	0.025 (2)	0.029 (2)	0.024 (2)	−0.002 (2)	−0.0009 (19)	−0.0056 (19)
C18	0.030 (3)	0.024 (2)	0.027 (2)	−0.002 (2)	−0.007 (2)	−0.0010 (19)
F1	0.0276 (16)	0.0464 (18)	0.0335 (16)	0.0017 (13)	0.0032 (13)	−0.0155 (13)
F2	0.0304 (17)	0.0502 (19)	0.0433 (18)	0.0012 (14)	0.0115 (14)	−0.0189 (15)
I1	0.02218 (17)	0.02611 (17)	0.01820 (16)	−0.00355 (12)	−0.00138 (12)	−0.00623 (11)
I2	0.02468 (18)	0.02723 (17)	0.02327 (17)	−0.00611 (13)	0.00320 (12)	−0.00869 (12)
O1	0.0204 (17)	0.0395 (19)	0.0243 (17)	0.0040 (14)	−0.0070 (14)	−0.0113 (14)
O2	0.0195 (16)	0.0316 (17)	0.0235 (16)	−0.0007 (13)	−0.0009 (13)	−0.0122 (14)

### Geometric parameters (Å, º)

**Table:**

C1—C6	1.374 (6)	C10—I2	2.094 (5)
C1—C2	1.386 (6)	C11—H11	0.950
C1—I1	2.085 (4)	C11—C12	1.385 (7)
C2—C3	1.367 (7)	C12—C13	1.377 (7)
C2—H2	0.950	C13—C14	1.389 (7)
C3—C4	1.382 (7)	C14—C15	1.385 (6)
C4—C5	1.378 (7)	C15—C16	1.519 (6)
C5—C6	1.394 (6)	C16—O2	1.437 (5)
C6—C7	1.514 (6)	C16—C18	1.519 (6)
C7—O1	1.437 (5)	C16—C17	1.525 (6)
C7—C8	1.521 (6)	F1—I1	2.045 (3)
C7—C9	1.523 (6)	F2—I2	2.046 (3)
C10—C15	1.372 (6)	I1—O1	2.022 (3)
C10—C11	1.382 (6)	I2—O2	2.017 (3)
			
C6—C1—C2	123.2 (4)	H11—C11—C10	121.3
C6—C1—I1	111.5 (3)	C13—C12—C11	120.3 (5)
C2—C1—I1	125.3 (4)	C12—C13—C14	120.6 (5)
C3—C2—C1	117.9 (5)	C15—C14—C13	120.4 (4)
H2—C2—C1	121.0	C10—C15—C14	117.3 (4)
C2—C3—C4	120.4 (5)	C10—C15—C16	118.2 (4)
C5—C4—C3	120.9 (4)	C14—C15—C16	124.5 (4)
C4—C5—C6	119.7 (5)	O2—C16—C15	107.5 (3)
C1—C6—C5	117.7 (4)	O2—C16—C18	110.3 (4)
C1—C6—C7	118.1 (4)	C15—C16—C18	109.6 (4)
C5—C6—C7	124.2 (4)	O2—C16—C17	106.0 (3)
O1—C7—C6	108.1 (4)	C15—C16—C17	111.9 (4)
O1—C7—C8	109.8 (4)	C18—C16—C17	111.3 (4)
C6—C7—C8	109.9 (4)	O1—I1—F1	166.40 (12)
O1—C7—C9	104.8 (4)	O1—I1—C1	80.58 (16)
C6—C7—C9	112.1 (4)	F1—I1—C1	86.21 (16)
C8—C7—C9	111.8 (4)	O2—I2—F2	166.81 (13)
C15—C10—C11	124.0 (4)	O2—I2—C10	80.30 (16)
C15—C10—I2	111.0 (3)	F2—I2—C10	87.13 (16)
C11—C10—I2	124.9 (4)	C7—O1—I1	113.7 (3)
C10—C11—C12	117.4 (5)	C16—O2—I2	113.6 (3)
			
C6—C1—C2—C3	0.7 (7)	C13—C14—C15—C10	−0.2 (6)
I1—C1—C2—C3	−178.5 (4)	C13—C14—C15—C16	177.5 (4)
C1—C2—C3—C4	−0.8 (7)	C10—C15—C16—O2	−22.0 (5)
C2—C3—C4—C5	−0.6 (8)	C14—C15—C16—O2	160.3 (4)
C3—C4—C5—C6	2.1 (7)	C10—C15—C16—C18	97.9 (5)
C2—C1—C6—C5	0.8 (7)	C14—C15—C16—C18	−79.8 (5)
I1—C1—C6—C5	−179.8 (3)	C10—C15—C16—C17	−138.1 (4)
C2—C1—C6—C7	177.8 (4)	C14—C15—C16—C17	44.3 (6)
I1—C1—C6—C7	−2.8 (5)	C6—C1—I1—O1	−11.4 (3)
C4—C5—C6—C1	−2.2 (7)	C2—C1—I1—O1	168.0 (4)
C4—C5—C6—C7	−179.0 (4)	C6—C1—I1—F1	171.9 (3)
C1—C6—C7—O1	21.8 (5)	C2—C1—I1—F1	−8.8 (4)
C5—C6—C7—O1	−161.3 (4)	C15—C10—I2—O2	13.5 (3)
C1—C6—C7—C8	−98.0 (5)	C11—C10—I2—O2	−167.8 (4)
C5—C6—C7—C8	78.8 (5)	C15—C10—I2—F2	−170.5 (3)
C1—C6—C7—C9	136.9 (4)	C11—C10—I2—F2	8.2 (4)
C5—C6—C7—C9	−46.3 (6)	C6—C7—O1—I1	−31.0 (4)
C15—C10—C11—C12	−0.3 (7)	C8—C7—O1—I1	89.0 (4)
I2—C10—C11—C12	−178.8 (3)	C9—C7—O1—I1	−150.7 (3)
C10—C11—C12—C13	0.0 (7)	F1—I1—O1—C7	38.2 (7)
C11—C12—C13—C14	0.2 (7)	C1—I1—O1—C7	24.4 (3)
C12—C13—C14—C15	−0.1 (7)	C15—C16—O2—I2	33.1 (4)
C11—C10—C15—C14	0.4 (6)	C18—C16—O2—I2	−86.4 (4)
I2—C10—C15—C14	179.0 (3)	C17—C16—O2—I2	153.0 (3)
C11—C10—C15—C16	−177.4 (4)	F2—I2—O2—C16	−44.7 (7)
I2—C10—C15—C16	1.2 (5)	C10—I2—O2—C16	−26.9 (3)
